# Removal of Phenols in Table Olive Processing Wastewater by Using a Mixed Inoculum of *Candida boidinii* and *Bacillus pumilus*: Effects of Inoculation Dynamics, Temperature, pH, and Effluent Age on the Abatement Efficiency

**DOI:** 10.3390/microorganisms9081783

**Published:** 2021-08-23

**Authors:** Daniela Campaniello, Barbara Speranza, Clelia Altieri, Milena Sinigaglia, Antonio Bevilacqua, Maria Rosaria Corbo

**Affiliations:** Department of Agriculture, Food, Natural Resources and Engineering (DAFNE), University of Foggia, Via Napoli 25, 71122 Foggia, Italy; daniela.campaniello@unifg.it (D.C.); Barbara.speranza@unifg.it (B.S.); milena.sinigaglia@unifg.it (M.S.); clelia.altieri@unifg.it (C.A.)

**Keywords:** phenols, *Candida boidinii*, *Bacillus pumilus*, table olive processing wastewater, bioremediation

## Abstract

The main goal of this paper was to assess the ability of a combination of *Candida boidinii* and *Bacillus pumilus* to remove phenol in table olive processing water, as a function of some variables, like temperature, pH, a dilution of waste and the order of inoculation of the two microorganisms. At this purpose *C. boidinii* and *B. pumilus* were sequentially inoculated in two types of table olive processing water (fresh wastewater, FTOPW and wastewater stored for 3 months-aged wastewater, ATOPW). pH (6 and 9), temperature (10 and 35 °C) and dilution ratio (0, 1:1) were combined through a 2^k^ fractional design. Data were modeled using two different approaches: Multifactorial Analysis of Variance (MANOVA) and multiple regression. A higher removal yield was achieved by inoculating *B. pumilus* prior to the yeast (192 vs. 127 mg/L); moreover, an increased efficiency was gained at 35 °C (mean removal of 200 mg/L). The use of two statistic approach suggested a different weight of variables; temperature was a global variable, that is a factor able to affect the yield of the process in all conditions. On the other hand, an alkaline pH could increase the removal of phenol at 10 °C (25–43%).

## 1. Introduction

The production of table olives is one of the most important activities in Spain, Greece and Italy, although other countries are gaining an increasing importance (Egypt, Turkey, Algeria, Syria, Morocco, United States, Argentina, Mexico and Peru). The annual world production of table olives is about 3 million tons [1]. A consequence of the high table olive production is the high quantity of wastewater; in fact, table olive processing requires some steps (treatment with lye and at least two washings) to remove the natural bitter taste of olives by using fresh water [2].

The composition and type of wastewater can be different, depending on the method used for processing table olives (e.g., Spanish-style, Greek style, Californian-style, etc.): wastewater from lye, washes and fermentation brines, or from washing the plant, cleaning the jars or containers, etc. [3]. Therefore, there are several components in TOPW (table olive processing wastewater), like sugars and phenolic compounds, nitrogenous compounds (especially amino acids), organic acids, tannins, pectins, carotenoids and oil residues; all these compounds are responsible of their polluting effect [4]. Phenols at high concentrations can only be degraded with difficulty because of their refractory nature or their toxicity towards many microorganisms [5]. The main phenolic compound in TOPW is hydroxytyrosol, which is the main product from the alkaline hydrolysis of oleuropein. Other moieties usually found in TOPW include tyrosol, 3,4-dihydroxyphenylacetic acid, 4-hydroxybenzoic acid, catechin, vanillic acid, vanillin, caffeic acid, p-coumaric acid, elenolide and luteolin 7-glucoside [5,6,7].

The adequate wastewater treatment and their controlled reuse in irrigation could contribute to water saving and to solve the water shortage issue complained in some countries in Mediterranean area; in fact, water scarcity represents a worrying menacing for the viability of agriculture [8]. However, the presence of phenols requires a careful control of the chemical composition as these contaminants may generate problems for agricultural production affecting crop quantity and quality [9]. Several treatments have been proved to be fairly effective in reducing the environmental impact of these wastewater, for example advanced oxidation processes such us Ozonation, Fenton reaction [5], Electrochemical treatment, TiO_2_ Photocatalysis, Electro-Coagulation, Wet Air Oxidation, but they are still too expensive or cannot always be applied at industrial level. Biological treatments, including aerobic and anaerobic processes according to the type of microorganisms and the presence or absence of oxygen, are considered as economical and effective alternative approaches [3,10,11].

Several studies report on the potentialities of some bacteria (*Bacillus*, *Pediococcus*, *Lactobacillus*, *Arthrobacter*, *Azotobacter*, *Pseudomonas* and *Ralstonia*) [12,13,14,15,16,17,18,19,20,21] and fungi (*Candida tropicalis*, *Candida cylindracea*, *Yarrovia lypolitica*, *Phanerodontia chrysosporium*, *Trametes versicolor*, *Funalia trogii*, *Lentinus edodes*, *Aspergillus niger* and *Aspergillus terreus*) [22,23,24,25,26,27,28,29,30,31,32] as bioremediation tools, although few data are available for TOPW. In the past, Campaniello et al. [10] evaluated the effect of a combination of *B. pumilus*/*C. boidinii* and *Trichoderma harzianum* on TOPW and found significant levels of COD (4000 mg/L for *T. harzianum* and 2400 mg/L for *C. boidinii*/*B. pumilus*) and phenol reduction (up to 1800 mg/L for the combination *C. boidinii*/*B. pumilus* and 2800 mg/L for *T. harzianum*). In that research, some variables were not studied, e.g., the protocol for inoculation of *Bacillus* and *Candida* and the kind of TOPW with respect of their “age”.

Therefore, the focus of this paper was on the use of the combination *B. pumilus*/*C. boidinii* to address the following aims:

(a) To evaluate if the way of inoculation (before yeast and then bacterium or *viceversa*) could affect the removal efficiency.
(b) To study the potentialities of the combination on the bioremediation of TOPW as a function of their age (TOPW treated immediately after their discharge or after a preliminary storage in temporary tanks, as usually done by producers).

The activities were done on phenols, as model compounds to assess the removal efficiency.

## 2. Materials and Methods

### 2.1. Microorganisms

*Bacillus pumilus* 13M and *Candida boidinii* 682, were used in this study [10]. *B. pumilus* and *C. boidinii* were grown before each assay in Tryptone Soya broth at 30 °C for 48 h (Oxoid, Basingstoke, UK) and on YPG broth at 25 °C for 72 h (yeast extract, 10 g/L; peptone, 20 g/L; glucose, 20 g/L; Oxoid), respectively.

### 2.2. Table Olive Processing Wastewater

Two types of TOPW were supplied by a factory located in Cerignola (Foggia county, Apulia, Italy): (a) TOPW collected immediately after their discharge (defined as fresh table olive processing wastewater, FTOPW; pH, 11.85; phenol, 315 mg/L; Chemical Oxygen Demand, 3000 mg/L); and (b) TOPW stored in settling tanks for at least three months (defined as aged table olive processing wastewater, ATOPW; pH, 6.43; phenols, 4.27 g/L; Chemical Oxygen Demand, 10,700 mg/L). Both the batches were from Spanish style processing.

### 2.3. Bioremediation through a Sequential Inoculation of Bacillus pumilus and Candida boidinii

The strains were harvested by centrifugation (1000× *g* for 10 min) and 6 log CFU/mL of both microorganisms were inoculated in FTOPW and ATOPW (the volume of each sample was 50 mL). Two types of experiments were performed: Test 1—initial inoculation of *C. boidinii* strain, followed (3 days after) by *B. pumilus*; and *viceversa* (Test 2).

Storage temperature (10 and 35 °C), pH (6.0 and 9.0; the pH of TOPW was adjusted through HCl or NaOH 1.0 N) and the dilution ratio with sterilized tap water (0, 1:1) were combined through a full 2^k^ design, while the type of wastewater (FTOPW and ATOPW) was used as an additional input factor. Table 1 shows the coded and tested values of the independent variables of the design (each combination was performed in both FTOPW and ATOPW). For each design abiotic controls (samples diluted with tap water and adjusted to different pHs) were prepared and analyzed as reported for samples with inoculated bacterium and yeast.

### 2.4. Microbiological and Chemical Analyses

Microbiological and chemico-physical analyses were done before inoculation, after 3 days (before the inoculation of the second microorganism) and after 8 days (end of the experiment). Viable count was evaluated on TSA + 0.17 g/L cycloheximide (Sigma-Aldrich, Milan, Italy) (30 °C for 24–48 h, *B. pumilus*) and on YPG agar + 0.1 g/L chloramphenicol (C. Erba, Milan, Italy) (25 °C for 48–72 h, *C. boidinii*); data were confirmed by random microscopic examination. pH was evaluated through a pH-meter Crison mod. 2001 (Crison instruments, Barcelona, Spain), while the concentrations of phenol were assessed by Folin–Ciocalteu protocol [33].

### 2.5. Statistical Analyses

The experiments were performed on two independent batches and repeated twice for each batch; data were modeled as reduction of phenols, compared to the initial concentration, both as actual concentration (mg/L) and as percentages (%). All values used for statistical purposes were corrected by subtracting the reduction in phenols in abiotic controls (1–2% after 8 days) from each combination.

The actual reduction values were used as input variables for a Multifactorial Analysis of Variance (MANOVA); the time of analysis (3 or 8 days), the combination of the design and the kind of inoculum were used as categorical predictors (qualitative independent variables). Statistical analysis was separately performed for FTOPW and ATOPW.

The second statistic was a multiple regression analysis through the option DoE (Design of Experiments) through the software Statistica for Windows, ver. 12.0 (Statsoft, Tulsa, OK, USA); the percentage of phenol reduction was the dependent variable while pH, temperature and dilution were the independent variables and the analysis was done separately for each time of sampling (3 or 8 days), kind of TOPW (fresh or aged) and way of inoculation (Test 1 or Test 2).

## 3. Results

### 3.1. Qualitative Effect of Sequential Inoculation, Sampling Time and Combination of pH/Temperature/Dilution

The first modeling was done for qualitative purposes to assess which were the significant variables able to affect the reduction of phenol concentration on TOPW; this analysis was done on actual values because the standardized data (percentages) could not address the basic rule of parametric statistic (homoscedasticity). In addition, since the initial concentration of phenols was different between fresh and aged TOPW, the results were separately analyzed.

Table 2 shows the standardized effects of individual and interactive terms of categorical predictors; all terms were significant, although with a different statistical weight. For both FTOPW and ATOPW, the most significant individual term was the kind of inoculum, followed by the combinations of the design and finally by time, while the factor inoculum/combination was the most significant interactive term.

The table of standardized effects offers a qualitative overview, but it does not report any quantitative information; details from this side could be obtained from the decomposition of the statistical hypothesis. The figures on the decomposition do not show actual values; they are a mathematical artifact and their goal is to show the effect of each categorical predictor by excluding all others.

**Figure 1:**
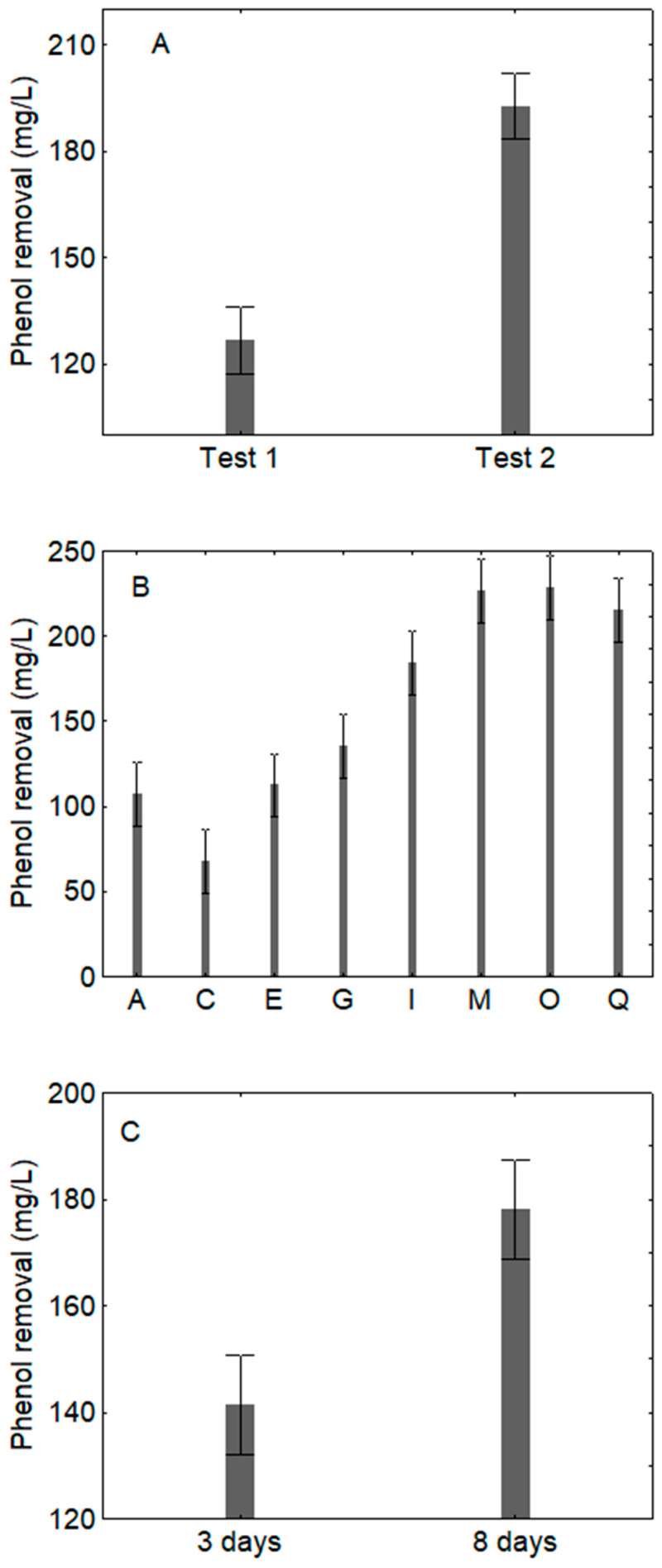
Decomposition of the statistical hypothesis for the effects of inoculation (**A**); *p*-level, < 0.0001), combinations of the design (**B**), *p*-level, < 0.0001) and time of sampling (**C**); *p*-level, < 0.0001) on the removal of phenols on FTOPW (fresh table olive processing water). Mean value ± 95% confidence interval; test 1: inoculum of *C. boidinii* followed 3 days after by *B. pumilus*; test 2: inoculum of *B. pumilus* and 3 days after *C. boidinii*.

For FTOPW, the best way for a higher removal efficiency was the use of bacterium and then yeast (test 2), corresponding to a mean efficiency of 192 mg/L vs. 127 mg/L for the other test (yeast and then bacterium) (Figure 1A). This figure was built by using all combinations of the design at both 3 and 8 days; therefore, it cannot be used to assess the removal efficiency for a combination or for a sampling time. It is a kind of mean efficiency of the protocol. In some combinations (Figure 1B), the removal efficiency was higher, that is in the batches I, M, O and Q, which were characterized by an incubation at 35 °C (mean value of ca. 200 mg/L). For this figure the only parameter assessed was the kind of combination, regardless the protocol (test 1 or test 2) or the sampling time. Finally, an incubation for 8 days caused a stronger removal efficiency (178 mg/L vs. 142 mg/L after 3 days) (Figure 1C).

**Figure 2:**
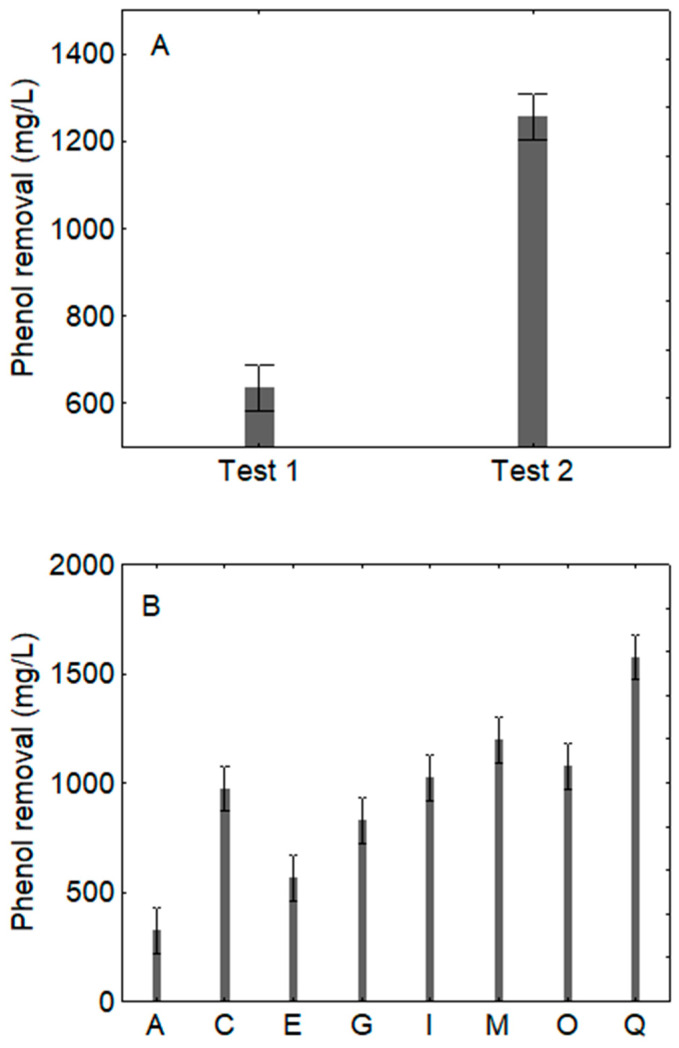
Decomposition of the statistical hypothesis for the effects of inoculation (**A**); *p*-level, <0.0001) and combinations of the design (**B**); *p*-level, <0.0001) on the removal of phenols on ATOPW (aged table olive processing water). Mean value ± 95% confidence interval. Test 1: inoculum of *C. boidinii* followed 3 days after by *B. pumilus*; test 2: inoculum of *B. pumilus* and 3 days after *C. boidinii*.

For ATOPW, the quantitative effect of the predictors was the same, although the actual values were higher due to a higher initial concentration of phenols: generally, test 2 caused a higher removal (1250 mg/L vs. 650 mg/L) (Figure 2A) and a strong reduction of phenols was found in the combinations I, M, O and Q (Figure 2B).

### 3.2. Mathematical Model

A modeling of phenol reduction was done through the DoE approach. This procedure is particularly useful for predictive purpose and to have quantitative details for some combinations of the factors not tested during the experiments [34].

The effects of pH, temperature and dilution ratio are in Table 3. Referring to the Test 1 (*C. boidinii* followed by *B. pumilus*), phenol removal was affected by pH as individual or quadratic term; temperature, as quadratic term (negative term), played a significant role in ATOPW (sample B, 8 days after). Finally, the interactions [pH] × [temperature] (as the negative term) and [temperature] × [dilution] were significant in ATOPW (sample A).

When *B. pumilus* was followed by *C. boidinii* (test 2) phenol reduction was affected by the quadratic terms of pH and temperature in ATOPW (sample F) and temperature, in FTOPW.

A second output of this approach was the development of polynomial equations, which mathematically described the quantitative correlation of significant variables versus the reduction of phenols.

The difference of this approach compared to MANOVA relies also on the background: for MANOVA all data were used and time was also used as a categorical predictor, therefore the statistical effects offer an overview on the global influence on phenol removal; on the other hand, for multiple regression a time-by-time approach was used and the statistic offers an overview for the effect of each variable for each time of sampling, as optimized by authors elsewhere and for other purposes [35].

**Figure 3:**
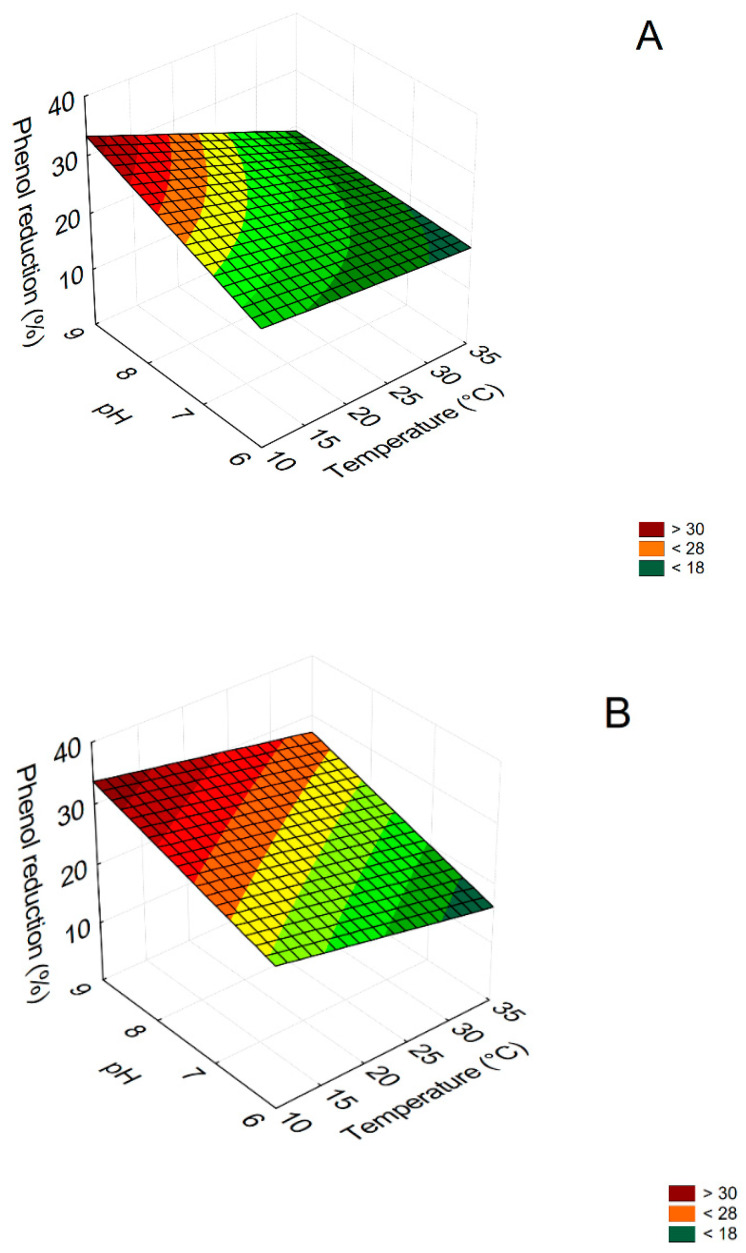
3D plots for the interaction pH/temperature on phenol reduction (test 1: inoculum of *C. boidinii* followed 3 days after by *B. pumilus*) in ATOPW (aged table olive processing water) after 3 (**A**) and 8 days after (**B**). Test A and B of Table 3.

For a quantitative estimation of the effects of the three parameters (pH, temperature and dilution), 3D plots were assessed through the polynomial equations. Figure 3 shows the interactions “pH by Temperature” in ATOPW when the inoculum of *C. boidinii* was followed by *B. pumilus* (test 1) after 3 and 8 days (samples A and B, respectively).

Figure 3A shows that after 3 days (before the inoculum of *B. pumilus*) the highest reduction of the phenolic content (>34%) was obtained at pH 8.5–9.0 and at 10–16 °C. After 8 days (Figure 3B), phenols were effectively reduced (>34%) at pH values between 8.5 and 9.0 and temperatures between 10–20 °C.

**Figure 4:**
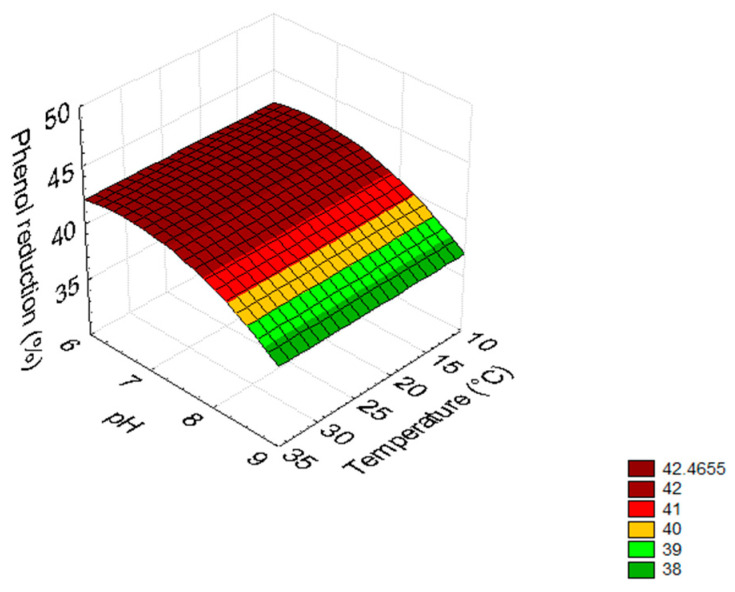
3D plots for the interaction pH/temperature on phenol reduction (test 1: inoculum of *C. boidinii* followed 3 days after by *B. pumilus*) in FTOPW (fresh table olive processing water) after 3 days. Test C of Table 3.

**Figure 5:**
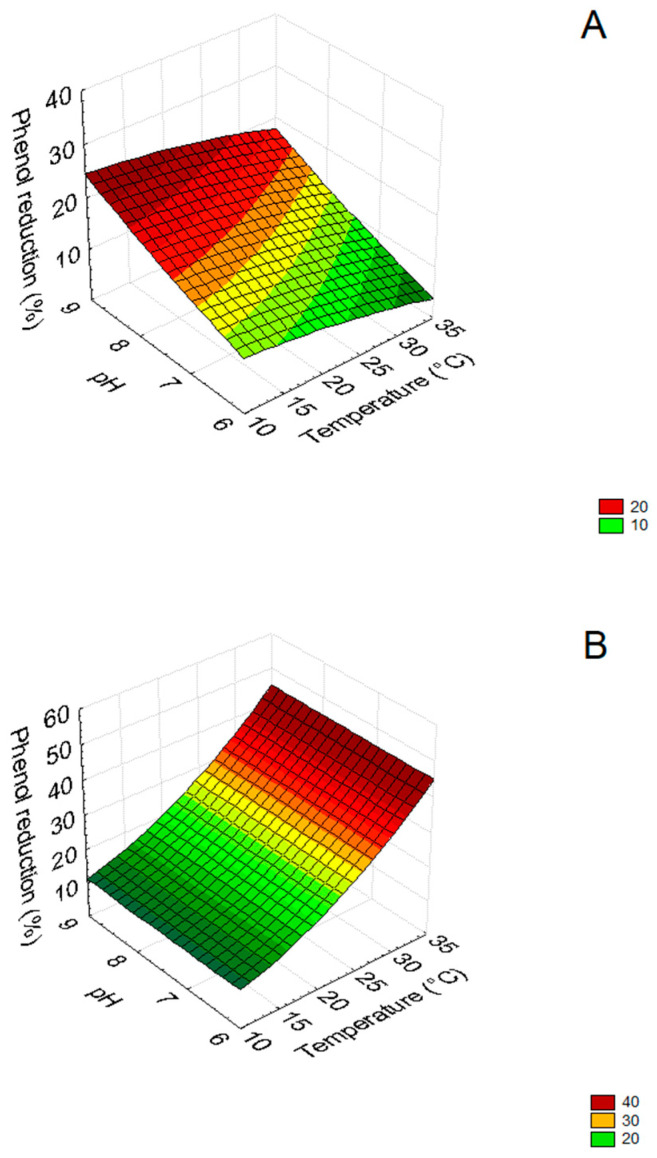
3D plots for the interaction pH/temperature on phenol reduction (test 2: inoculum of *B. pumilus* followed 3 days after by *C. boidinii*) in ATOPW (aged table olive processing water) (**A**) and FTOPW (fresh table olive processing water) (**B**) after 8 days. Test F and H of Table 3.

Concerning FTOPW the phenol degrading ability of the tested strains (test 1) were affected by pH; in fact, after 3 days, the maximum reduction of phenols (>42%) was obtained for pH values between 6 and 7 and regardless the applied temperature (Figure 4). A similar phenol reduction (>43%) was observed after 8 days at alkaline pH values (data not shown). The results for test 2 (*B. pulimus* followed by the yeast) after 8 days are in Figure 5. As expected by the standardized effects and the polynomial equations, phenol removal was affected by pH and/or temperature. In the case of ATOPW, the highest phenol reduction (25%) was found at pH 9 and at 10 °C, due to the mathematical positive term of pH and the negative effect of temperature (Figure 5A). On FTOPW, the highest phenol removal (45%) was found at 35 °C, regardless pH (Figure 5B)

Table 4 shows the viable count of *B. pumilus* and *C. boidinii* after 8 days. In test 2, *B. pumilus* attained higher counts (7.16–7.58 log CFU/mL) due to the longer incubation period (8 days), while in test 1 the bacterium was at ca. 6 log CFU/mL. The viable counts of yeast were ca. 5 log CFU/mL with some exceptions to this generalized statement.

## 4. Discussion

The management of TOPW, similarly to oil mill wastewater, represent a serious problem. Some Mediterranean countries try to “solve” this problem by storing TOPW in open evaporation ponds, but malodorous gases can be produced by putrefactive or methanogenic bacteria, thus leading to waste leakage and migration into groundwater and deep soil and consequently, to probable contamination events [36]. An alternative approach can be the use of physical, physico-chemical, thermal and oxidation methods that convert TOPW into by-products; however, they have the disadvantage of generating secondary streams containing pollutants that need additional treatments and high energy costs [36].

Phenols represent one of the major pollutants contained in wastewater; they are highly hazardous and exerts a corrosive and an irritation effect [37] and according to US Environmental Protection Agency their critical thresholds are 0.5 mg/L (wastewater discharge) and 1 mg/L (surface water and sewerage system) [37]. The polluting effect of phenols could be reduced through their degradation into simple end products by fungi and bacteria; this approach is known as bioremediation [38]. Generally, these microorganisms are isolated from a similar source type in a dissimilar or similar ecosystem, and follow a variety of metabolic pathways for the degradation of the pollutant compounds [37].

The strains used in this paper have been proposed in the past for an effective bioremediation of both TOPW and olive mill wastewater [10,39,40]; the combination of the two strains was studied regardless the protocol for inoculation and the age of TOPW. The potentialities of *B. pumilus* and *C. boidinii* probably rely on their ability to degrade some phenols into by-products (for example, protocatechuic acid and caffeic acid for the bacterial strain) or to be absorbed into surfaces, as also stated elsewhere [10,12].

Phenol removal/degradation relies on many factors (pH, temperature and others); therefore, it is important the choose the optimal combinations of factors to improve the efficienvy of the process and to reduce its cost. Patil and Jena [37] studied the effect of initial phenol concentration, pH, temperature, inoculum size and concentration of medium components) and found that *B. pumilus* strain OS1 achieved the maximum phenol degradation (99.99%) at pH 7.07, temperature 29.3 °C, inoculum size 6.3% (*v/v*), (NH_4_)_2_SO_4_ 392.1 mg/L. On the other hand, in the past, the removal efficiency of *C. boidinii* in olive mill wastewater (that is in a different matrix) and as a cocktail with other strains, was affected by (NH_4_)_2_SO_4_ and temperature, with a maximum at 30 °C. Other strains of *C. boidinii* were also reported to eliminate 40% of the phenolic compounds in crude olive mill wastewater [41].

Focusing on the results of this research, the traits of the strains could explain the higher removal efficiency in some combinations (I, M, O and Q); the microorganisms, in particular *B. pumilus*, are mesophilic strains and their metabolism is enhanced at 30–35 °C. The effect of temperature was also found in the time-by-time modeling (static approach) for some times of sampling; on the other hand, pH was found as a significant predictor only through the multiple regression, thus suggesting that its effect was not global but it acts only in some conditions (for example for lower incubation periods). As stated elsewhere [14], alkaline pH values enhanced phenol removal and this effect could be probably attributed to a shift of the charges on the surface and to an enhanced enzymatic activity.

The last important result was the fact that the protocol for the inoculation could strongly affect the removal, as generally at higher temperature it is important to inoculate before bacterial strain and then yeast. This result was probably due to the enhanced metabolism of *B. subtilis* at 35 °C, while at this temperature preliminary results showed that *C. boidinii* experienced a reduced growth rate. The viable count of *B. pumilus* was generally affected by the protocol, as in test 2 after 8 days the counts of bacterium were generally higher. Some preliminary experiments revealed a stable microbial consortium and a higher performance when the microorganisms were used as a cocktail; however, further assays are required to elucidate the mode of action for phenol removal and how the microorganisms could collaborate for this purpose.

Finally, a focus on the age of TOPW and their effect on the initial content of phenols; in the current work, ATOPW had higher amounts of phenols, due to a probable chemical and photocatalytic degradation of phenols at high molecular weight [42]; it is a matter of debate if for a producer is convenient to store TOPW or to immediately proceed to their bioremediation.

## 5. Conclusions

In conclusion, it is well known that the use of yeasts and bacterial strains for the bioremediation of OMW is a promising way and as result in this paper *B. pumilus* and *C. boidinii* were successfully used for the bioremediation of TOPW.

The use of two statistic approaches showed that there were some global effects, where for global we mean significant variables for the whole process and variables significant only for a limited time: temperature is a global variable, while pH was probably a limited variable. The removal efficiency was higher at 35 °C (mean removal, 200 mg/L), while pH acted only in some conditions (for example at 10 °C), with an increased efficiency under alkaline combinations (at pH 9 reduction of phenol by 24–43%).

Another variable affecting the efficiency was the way of inoculation: in a sequential inoculation the order is important and the efficiency was higher when *B. pumilus* was the first to be inoculated.

## Figures and Tables

**Table 1:** Combinations of the design.

	Coded Values			Actual Values		
Run	pH	Dilution	Temperature	pH	Dilution	Temperature
A	−1	−1	−1	6	0	10 °C
C	+1	−1	−1	9	0	10 °C
E	−1	+1	−1	6	1:1	10 °C
G	+1	+1	−1	9	1:1	10 °C
I	−1	−1	+1	6	0	35 °C
M	+1	−1	+1	9	0	35 °C
O	−1	+1	+1	6	1:1	35 °C
Q	+1	+1	+1	9	1:1	35 °C

**Table 2:** Standardized effects of MANOVA. ATOPW, aged table olive processing water; FTOPW, fresh table olive processing water.

	FTOPW	ATOPW
Inoculum (test 1 or test 2)	98.778	279.971
Combination of the design	43.787	53.440
Time of sampling	30.514	45.614
Inoculum vs. combination	69.665	93.794
Inoculum vs. time	4.656	9.749
Combination vs. time	3.170	3.732
Inoculum vs. combination vs. time	11.416	4.599

**Table 3:** Effect of pH, temperature and dilution ratio on phenol reduction by *Bacillus pumilus* and *Candida boidinii* sequentially inoculated (test 1 and test 2) in ATOPW and FTOPW. R^2^, determination coefficient; R^2^_adjusted_, determination coefficient adjusted for multiple regression; F-test, Fisher test value; SE, standard error of the model.

	Test 1				Test 2			
	Inoculum of *C. boidinii* Followed by *B. pumilus*				Inoculum of *B. pumilus* Followed by *C. boidinii*			
	A	B	C	D	E	F	G	H
pH	11.845	14.0612	7.198	12.269	-	-	-	-
Temperature	-	-	-	-	-	-	-	-
Dilution	-	-	-	-	-	-	-	-
pH^2^	-	-	−4.448	−6.699	-	8.331	-	-
Temperature^2^	-	−2.488	-	-	-	−2.40	3.013	6.209
Dilution^2^	-	-	-	-	-	-	-	-
pH × temperature	−5.601	-	-	-	-	-	-	-
pH × dilution	-	-	-	-	-	-	-	-
T × dilution	3.123	-	-	-	-	-	-	-
R^2^	0.993	0.983	0.980	0.997	-	0.941	0.982	0.865
R^2^_adjusted_	0.985	0.977	0.973	0.994	-	0.921	0.971	0.843
F-test	133.52	171.79	148.55	517.29	-	47.515	91.522	38.556
*p*-level	0.0016	0.00001	0.0001	0.0000	-	0.0021	0.00009	0.00081
SE	2.906	3.954	6.502	3.152	-	4.650	5.933	7.811
	Equations							
A	R = 4.404 × pH − 0.08 × pH × temperature + 0.452 × temperature × dilution							
B	R = 3.870 × pH − 0.006 × temperature^2^							
C	R = 12.803 × pH − 0.965 × pH^2^							
D	R = 11.241 × pH − 0.718 × pH^2^							
E	-							
F	R = 0.311 × pH^2^ − 0.006 × temperature^2^							
G	R = 0.06 × temperature^2^							
H	R = 8.274 + 0.03 × temperature^2^							

A, B = ATOPW; inoculum of *C. boidinii* followed, 3 days later, by *B. pumilus* (data at 3 days, A; data at 8 days, B); C, D = FTOPW; inoculum of *C. boidinii* followed, 3 days later, by *B. pumilus* (data at 3 days, C; data at 8 days, D).; E, F = ATOPW; inoculum of *B. pumilus* followed 3 days later, by *C. boidinii* (data at 3 days, E; data at 8 days, F).; G, H = FTOPW; inoculum of *B. pumilus* followed 3 days later, by *C. boidinii* (data at 3 days, G; data at 8 days, H).

**Table 4:** Viable count (log CFU/mL) of *B. pumilus* (13M) and *C. boidinii* (682) sequentially inoculated (test 1: inoculum of *C. boidinii* followed by *B. pumilus*; test 2: inoculum of *B. pumilus* followed by *C. boidinii*) in ATOPW and FTOPW. Inoculum, 6.20 log CFU/mL for *B. pumilus* and 6.15 log CFU/mL for *C. boidinii*. Data are the average of two replicates.

	FTOPW				ATOPW			
	Test 1		Test 2		Test 1		Test 2	
	13M	682	13M	682	13M	682	13M	682
A	5.91	5.76	7.17	5.34	5.75	6.59	7.26	5.26
C	6.26	5.49	7.78	5.28	5.91	5.07	7.32	5.71
E	6.31	6.83	7.13	5.32	5.68	5.76	7.53	5.71
G	6.02	5.71	7.81	5.60	6.14	5.63	7.54	5.57
I	5.71	5.49	7.72	4.48	5.86	4.20	7.15	5.15
M	6.17	5.34	7.40	4.15	5.85	4.32	7.41	3.91
O	6.03	4.84	7.73	4.32	5.94	4.40	7.66	5.08
Q	6.05	6.86	7.58	4.00	6.04	6.03	7.54	5.85

## Data Availability

Raw data are available upon request; the relevant information and details are in the article.
